# Robust Heartbeat Classification for Wearable Single-Lead ECG via Extreme Gradient Boosting

**DOI:** 10.3390/s21165290

**Published:** 2021-08-05

**Authors:** Huaiyu Zhu, Yisheng Zhao, Yun Pan, Hanshuang Xie, Fan Wu, Ruohong Huan

**Affiliations:** 1 College of Information Science and Electronic Engineering, Zhejiang University, Hangzhou 310027, China; zhuhuaiyu@zju.edu.cn (H.Z.); zhaoys@zju.edu.cn (Y.Z.); 2 R&D Department, Hangzhou Proton Technology Co., Ltd., Hangzhou 310012, China; hanshuang.xie@protontek.com (H.X.); fan.wu@protontek.com (F.W.); 3 College of Computer Science and Technology, Zhejiang University of Technology, Hangzhou 310023, China; huanrh@zjut.edu.cn

**Keywords:** heartbeat classification, single-lead ECG, ECG database, wearable, XGBoost

## Abstract

Wearable electrocardiogram (ECG) monitoring devices have enabled everyday ECG collection in our daily lives. However, the condition of ECG signal acquisition using wearable devices varies and wearable ECG signals could be interfered with by severe noises, resulting in great challenges of computer-aided automated ECG analysis, especially for single-lead ECG signals without spare channels as references. There remains room for improvement of the beat-level single-lead ECG diagnosis regarding accuracy and efficiency. In this paper, we propose new morphological features of heartbeats for an extreme gradient boosting-based beat-level ECG analysis method to carry out the five-class heartbeat classification according to the Association for the Advancement of Medical Instrumentation standard. The MIT-BIH Arrhythmia Database (MITDB) and a self-collected wearable single-lead ECG dataset are used for performance evaluation in the static and wearable ECG monitoring conditions, respectively. The results show that our method outperforms other state-of-the-art models with an accuracy of 99.14% on the MITDB and maintains robustness with an accuracy of 98.68% in the wearable single-lead ECG analysis.

## 1. Introduction

Arrhythmia refers to any changes of the normal electrocardiography (ECG) signals, that is, the electrical impulses causing abnormal heart rhythms, which are characterized by transience, paroxysm, and usually with no obvious symptoms [1]. In the field of arrhythmia detection, beat-level arrhythmia analysis based on everyday ECG signals has become a valuable and promising technique for the prevention and early detection of patients with arrhythmias [2]. With the innovation of mobile health technologies, clinical-level wearable ECG monitoring devices with limited lead channels have been designed in a variety of physical forms, e.g., card-type [3], watch-type [4], and patch-type [5] and applied in dedicated clinical diagnosis and treatment scenes like immediate real-time monitoring and ultra-long-term monitoring. These devices are now becoming the main source of everyday ECG signals gradually.

However, as we could obtain massive ECG signals from wearable ECG monitoring devices nowadays, it remains a challenging task for the computer-aided automated analysis of arrhythmia based on ECG signals [6]. This is due to the fact that the condition of ECG signal collection is commonly different and wearable ECG signals could be interfered with by severe noises, especially for those ECG signals collected using wearable single-lead ECG monitoring devices deployed in the environment of daily life usage. Automated wearable single-lead ECG signal analysis is of great significance to the monitoring of everyday cardiac activity for the detection of abnormal heart conditions, in which case human monitoring and interpretation are not feasible for the real-time or ultra-long-term ECG signal diagnosis requirement regarding the timeliness, efficiency, operability, and even accuracy [2].

Heartbeat classification, i.e., the beat-level ECG analysis, is the most common way for automated arrhythmia diagnosis [7]. The generic pipeline of the machine-learning-based beat-level ECG analysis includes signal denoising, heartbeat detection, handcrafted feature extraction, and heartbeat classification. For deep-learning-based methods, the handcrafted feature extraction could be replaced by the data-driven deep feature extraction, and the given data are usually the beat-by-beat ECG fragments instead of the complete ECG sequences. Since every cardiac cycle could be diagnosed through beat-level ECG analyzation and annotation, the heartbeat classification is the most widely applied method for computer-aided automated ECG analysis. Yet, most methods perform well on ECG datasets with conventional static ECG signals, while they suffer from the analysis of wearable single-lead ECGs.

In this paper, we propose an extreme gradient boosting (XGBoost) [8] based beat-level ECG analysis method with new handcraft features for a robust automated diagnosis of wearable single-lead ECG. We design a set of five-dimensional morphological features regarding QRS complexes and RR intervals, as well as some wavelet coefficient characteristics, to build our feature vector for highly efficient heartbeat classification, and we divide all heartbeats into five classes, i.e., supraventricular ectopic beats (S), ventricular ectopic beats (V), the fusion of ventricular and normal beats (F), paced beats (Q), and other types of heartbeats (N), referring to the standard of the Association for the Advancement of Medical Instrumentation (AAMI) [9].

**Figure 1:**
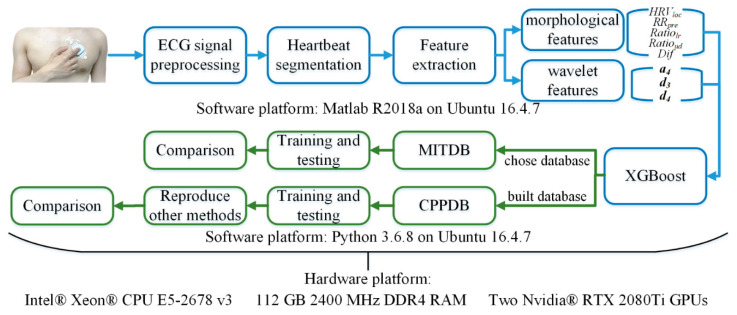
The overview framework of our study, including the framework of the proposed method (blue) and the overview of the conducted experiment (green).

We adopted the widely used MIT-BIH Arrhythmia Database (MITDB) [10] to evaluate the performance of beat-level ECG analysis and compare our method with other state-of-the-art models. Moreover, we built a wearable single-lead ECG database with the beat-level annotation using a clinical-level patch-type ECG device approved by the National Medical Products Administration (NMPA) of China, to evaluate different methods in the realistic wearable ECG monitoring scenario. The results showed that our method achieved the highest accuracy in both the static and wearable ECG analysis tasks among the state-of-the-art methods. An overview of our study is shown in Figure 1.

The main contributions of this paper are summarized as follows.

1. We proposed three novel morphological features, which form an effective morphological feature set with two well-used morphological features. Further, we combined a morphological feature set and wavelet coefficient characteristics as the handcraft features of XGBoost to achieve the best performance on both databases;
2. We built a dedicated ECG database to evaluate the performance of the proposed heartbeat classification method, as well as other state-of-the-art methods, especially for the accuracy analysis of these methods on wearable single-lead ECG signals under an everyday ECG monitoring scenario.

## 2. Related Work

### 2.1. Arrhythmia Classification Methods

In the recent past, researchers have attempted to classify arrhythmias on the heartbeat level using many different machine learning algorithms, such as K-nearest neighbors classifiers [11], the fuzzy clustering neural network [12], the adaptive neural network [13], decision trees [14], Bayesian classifiers [15], the support vector machine (SVM) [16], the convolutional neural network (CNN) [17,18,19], and the deep residual network (ResNet) [20].

For those handcrafted feature-based methods, Jekova et al. [11] applied the K-nearest neighbors classifier with features including the maximum peak/valley amplitude, the peak/valley area, RR intervals, the slope of QRS, etc., to classify the heartbeats. Özbay et al. [12] divided the ECG signals into 200-point heartbeat fragments and sent them to the fuzzy clustering neural network for a ten-class heartbeat classification with the hidden layer optimization. Barro et al. [13] created heartbeat templates based on the morphological characteristics of the heartbeats, and sent them to an adaptive neural network for multi-lead ECG heartbeat classification. This network could adaptively evaluate the signal quality of each input lead and select the best lead signal as the main basis for the classification. Mohanty et al. [14] performed a five-class heartbeat classification by extracting a 13-dimensional feature vector of ECG signals and utilizing the C4.5 decision tree method; Marinho et al. [15] combined the Fourier transform, Goertzel algorithm, higher-order statistics, and the structural co-occurrence matrix for feature extraction and analyzed heartbeats using the Bayesian classifier to achieve a highly efficient classification. Lastly, Mondéjar-Guerra et al. [16] applied the product rule to fuse SVMs for each type of feature and carry out the final classification result.

Several studies also introduced deep learning methods into ECG analysis. Zhai et al. [17] transformed the ECG beats into a dual-beat coupling matrix as two-dimensional inputs to the CNN (2D CNN) and classified the heartbeats into five categories. Golrizkhatami et al. [18] combined the multi-stage CNN features and the handcrafted features with a decision-level fusion using three classifiers to achieve the five-class heartbeat classification. Romdhane et al. [19] applied two CNN blocks with the focal loss function and RR interval-related segmentation to improve the classification task. Li et al. [20] used ResNet to process 2-lead ECG signals in combination and achieved a high heartbeat analysis performance.

In general, the heartbeat classification based on traditional machine learning methods such as K-nearest neighbors and SVM still has certain room for improvement in classification performance, while deep learning methods such as CNN have better performances as the classifier yet bring in heavy calculation and training time costs at the same time. Meanwhile, most studies continue to merely use ECG databases with good signal quality, e.g., the MITDB, to evaluate the performance of heartbeat classification. However, the situation could be different when facing ECG signals in wearable conditions.

### 2.2. Database for Evaluating Classification Methods

Due to the unbalanced development of traditional databases and dynamic databases, most published heartbeat classification methods used the following public standard ECG database:

1. The MIT-BIH database (MITDB) [10,21] is the first widely referenced database for arrhythmia classification. This database provides 48 two-channel half-hour ECG recordings (360 samples per second), which were annotated by at least two cardiologists;
2. The MIT-BIH Atrial Fibrillation database (AFDB) [21] includes 25 two-channel ECG records of subjects with atrial fibrillation. Each ECG record (one per patient) lasts 10 h and the sampling frequency is 250 samples per second;
3. The St. Petersburg Institute of Cardiological Technics (INCART) [21] contains 75 half-hour ECG recordings, extracted from 32 Holter recordings (one per patient). The sample rate for the 12-lead ECGs is 257 samples per second;
4. The AHA database [22] can be obtained after paying a fee on the emergency care Research Institute website. This database contains 155 recordings covering 8 types of arrhythmias. Each original ECG record (one per patient) lasts 3 h in total and was divided in periods of at least half an hour. Twelve lead ECGs were collected with a sampling frequency of 250 samples per second and 12 bits of precision;
5. The Physikalisch-Technische Bundesanstalt database (PTB) [21,23] includes 549 15-channel (12 + 3 Frank-lead) ECG recordings, digitized at 10 kHz per channel with 16-bit resolution. These recordings were obtained from 290 subjects in the age range of 17–87 years;
6. The supraventricular arrhythmia database (SUPRA) [21] consists of 78 half-hour ECG recordings obtained from 78 patients who experienced supraventricular arrhythmia. The information was digitized in twelve ECG channels with 125 samples per second and 12 bits of precision;
7. The PHYSIOBC [24] is a new database built in Mexico. This database contains 182 ECG records of 91 patients, ranging in age from 18 to 70.

For the dynamic database, relevant studies have been published in recent years; however, there remains no public, wearable ECG database. Shen et al. [25] used a limb two-lead wearable device to collect real-time ECG data and built a wearable ECG database. The authors claimed that 2000 30-s records were collected from more than 200 subjects diagnosed with heart disease.

## 3. Materials and Methods

The proposed XGBoost method shown in the blue box of Figure 1 can realize fast and effective automatic heartbeat classification. We first preprocess the ECG signal containing various noises to improve the signal quality, and divides the ECG signal into heartbeat fragments according to the cardiac cycle; then, we extract the morphological features of each heartbeat fragment and perform discrete wavelet decomposition to obtain the wavelet coefficient features, generate heartbeat feature vectors, and use XGBoost classifiers to train the beat-level classification models; finally, the trained model can automatically divide heartbeats into five AAMI categories, i.e., N, S, V, F, and Q.

### 3.1. Signal Preprocessing

The original raw ECG signals, especially wearable ECGs, commonly contain a variety of noises, such as baseline wandering, power frequency interference, electromyography (EMG) interference, etc. Signal denoising could contribute to improve the signal-to-noise ratio and reduce the impact of noises on beat-level classification. In this study, we use the method we proposed in [26] to preprocess the ECG signals and detect the R-peak. Several techniques such as de-averaging, median filtering, and finite impulse response (FIR) filtering are applied to perform primary denoising, as shown in Algorithm 1. Then, the Kalman filter is used to suppress the EMG noises of ECG signals, while maintaining the QRS regions for the R-peak detection. The R-wave peak positions are further determined by the wavelet-based method [27] and used to extract heartbeat fragments of each cardiac cycle in ECG signals.

**Algorithm** **1** The proposed ECG preprocessing algorithm: Primary Denoising
**Symbol setting:**
sr: the sample rate of ECG signals$y_{r}$: raw ECG signals$y_{f}$: filtered ECG signals$a_{median}$: the parameter of the median filter$f_{l}/f_{u}\;\&\;f_{order}$: the lower/upper cut-off frequency and order of the band-pass FIR filter$beta_{Kaiser}$: the parameter of the Kaiser windowmean(): mean functionround(): rounding function
**Process:**
A. Remove the direct current (DC) component and baseline drift in ECG signals    Step 1: DC component removing: $y_{1}=y_{r}-mean\left(y_{r}\right)$    Step 2: Preliminary removal of the baseline wandering:      **if**
$round\left(a_{median}\times sr\right)$** is an odd number,**        $p_{median}=round\left(a_{median}\times sr\right)$;      **else**        $p_{median}=round\left(a_{median}\times sr\right)+1$;      **end**      Perform $p_{median}$ points median filtering on $y_{1}$ to obtain $y_{2}$;B. Filter ECG signals:    Step 3: Construct the FIR filter according to $f_{l}$, $f_{u}$ and $f_{order}$;    Step 4: Calculate the Kaiser window according to $f_{order}$ and $beta_{Kaiser}$;    Step 5: Apply the windowed FIR filter to process $y_{2}$ and obtain $y_{3}$;C. Further removal of the baseline drift    Step 6: Perform $p_{median}$ points median filtering on $y_{3}$ to obtain $y_{f}$.

### 3.2. Feature Extraction

Many heartbeat features including morphological features and wavelet features were proposed [28,29,30] and made different levels of contribution to the beat-level classification performance. In order to facilitate our feature extraction of ECGs in MITDB, extracted heartbeat fragments are truncated to 151 sample points, including 50 sample points before the R-peak point, the R-peak point itself, and 100 sample points after the R-peak point. Note that we choose the input sample points, the parameters in feature extraction, and write the experimental code according to the sampling frequency of the most referenced MITDB, which is 360 Hz. When the sampling frequency of the input signal is not 360 Hz, we would resample it to 360 Hz or scale the input sample points and the parameters we provided in this paper.

Morphological features and wavelet features are extracted from each heartbeat fragments. We use a total of five morphological features, including the well-used previous RR interval, i.e., $RR_{pre}$ and the local heart rate variability $HRV_{loc}$ as in Equation (1):

(1) $$HRV_{loc}=RR_{pos}-RR_{pre}$$

where $RR_{pos}$ is the RR interval of the next heartbeat of the current one. In addition, we present three novel morphological features, i.e., the area ratio of the left and right sides of the R-peak, i.e., $Ratio_{lr}$, the area ratio of the above and below the fiducial line, i.e., $Ratio_{ud}$, and the amplitude difference within the duration of 220 ms, where the ratio of the duration before R-wave peak to the duration after R-wave peak is 3:5, i.e., Dif, as in Equations (2)–(4), where hb(i) is the amplitude of the $i^{th}$ point of a heartbeat fragment.

(2) $$Ratio_{lr}=\sum_{i=1}^{50}hb\left(i\right)/\sum_{i=52}^{151}hb\left(i\right)$$

(3) $$Ratio_{ud}=\sum hb\left(i\right)>0/\sum hb\left(i\right)<0$$

(4) Dif=max[hb]−min[hb],hb={hb(i)| i∈[21,101]}

For wavelet features of ECG heartbeats in MITDB, we used the combination of approximation coefficients at level 4, i.e., $a_{4}$, and detail coefficients at levels 3 and 4, i.e., $d_{3}$ and $d_{4}$, with the wavelet decomposition filter bank as shown in Equations (5) and (6).

(5) $$Lo_{D}=\left[0.000,\;0.125,\;0.375,\;0.375,\;0.125,\;0.000\right]$$

(6) $$Hi_{D}=\left[-0.0061,-0.0868,-0.5798,\;0.5798,\;0.0868,\;0.0061\right]$$

These 51 coefficients are thus derived as the wavelet features for each heartbeat. Finally, the morphological features and the wavelet decomposition coefficient features are built as a 56-dimensional feature vector, which is used for the XGBoost heartbeat classification training process.

### 3.3. XGBoost Classifier

XGBoost belongs to the Boosting family, which is fast and can be accelerated by parallel computing. The objective function of the XGBoost is shown in Equation (7):

(7) $$Obj^{\left(n\right)}=\sum_{m=1}^{M}L\left(y_{m},{\hat{y}}_{m}^{\left(n-1\right)}+f_{n}\left(x_{m}\right)\right)+\Omega \left(f_{n}\right)+constant$$

where L is the training loss function, ${\hat{y}}_{m}^{\left(n-1\right)}$ is the prediction result of the previous n−1 trees, $f_{n}\left(x_{m}\right)$ is the prediction result of the $n^{th}$ tree; β Ω is a regularization term. The definition of $\Omega \left(f_{n}\right)$ is as Equations (8) and (9):

(8) $$\Omega \left(f_{n}\right)=\gamma T+\frac{1}{2}\lambda \sum_{t=1}^{T}\omega_{t}^{2}$$

(9) $$f_{n}\left(x\right)=\omega_{q\left(x\right)}$$

where γ and λ are the pseudo-regularization hyperparameter that controls the complexity of the model and helps to avoid the overfitting, T is the number of leaves, $\omega_{t}^{2}$ is the L2 norm for leaf weights ω, which is the classification category in the classification problem, and q is the mapping relationship between each sample value and the leaf node, representing the structure of the tree.

Removing the constant in Equation (7) and applying the Taylor expansion approximation of the loss function, it becomes Equation (10):

(10) $$Obj^{\left(n\right)}=\sum_{m=1}^{M}\left[g_{m}f_{n}\left(x_{m}\right)+\frac{1}{2}h_{m}f_{n}^{2}\left(x_{m}\right)\right]+\Omega \left(f_{n}\right)$$

where $g_{m}$ and $h_{m}$ are the first- and second-order gradients of the loss function, respectively, as in Equations (11) and (12):

(11) $$g_{m}=\partial_{{\hat{y}}^{\left(n-1\right)}}L\left(y_{m},{\hat{y}}^{\left(n-1\right)}\right)$$

(12) $$h_{m}=\partial_{{\hat{y}}^{\left(n-1\right)}}^{2}L\left(y_{m},{\hat{y}}^{\left(n-1\right)}\right)$$

Furthermore, we could derive the final objective function by parameterizing the regularization term and introducing the tree structure q(x), as in Equation (13):

(13) $$Obj^{\left(n\right)}=-\frac{1}{2}\sum_{t=1}^{T}\frac{{\left(\sum_{m\in I_{t}}g_{m}\right)}^{2}}{\sum_{m\in I_{t}}h_{m}+\lambda }+\gamma T$$

where $I_{t}=\{m|q\left(x_{m}\right)=t\}$ is the region defined by the leaf node T. The training process could make full use of the advantages of parallel computing to realize fast model training, since the XGBoost optimized its performance on multi-core CPUs.

### 3.4. Evaluation Metrics

In this paper, the sensitivity (Se), precision (+P), accuracy (Acc), and *F*1 score (F1) were derived for the classification performance evaluation of methods, as in Equations (14)–(17).

(14) Se=TP/(TP+FN)×100%

(15) +P=TP/(TP+FP)×100%

(16) Acc=(TP+TN)/(TP+TN+FP+FN)×100%

(17) F1=2×TP/(2×TP+FP+FN)×100%

where TP is the number of true positive cases, TN is the number of true negative cases, FP is the number of false positive cases, and FN is the number of false negative cases.

### 3.5. Study Population

In this paper, both the widely used MITDB [10] and a self-collected wearable single-lead ECG database were collected using the NMPA-cleared CarePatch™ ECG patch (NMPA#ZJ20202070050) were adopted for performance evaluation.

#### 3.5.1. MITDB

The MITDB is a dataset of standard test material used for the evaluation of arrhythmia detectors and classifiers since 1980 in innumerable scientific works. It contains 48 2-channel ECG signals of 30 min, with a sample rate of 360 Hz and a resolution of 11 bits. According to the AAMI standard [9], we mapped the MITDB heartbeat classes to the AAMI classes and divided them into the training set DS1 and the test set DS2 as described in Table 1 [31] to make a fair comparison with other state-of-the-art methods. The label mapping rule and the heartbeat distribution of each class are shown in Table 2.

#### 3.5.2. CarePatch™ ECG Patch Database

Since wearable ECG monitoring suffers from disturbances introduced by people’s daily activities, the long-term single-lead ECGs collected by wearable devices usually contain more heartbeat patterns and noise interference during many days of continuous wearing than resting ECGs. As wearable ECGs put forward higher requirements on the ECG analysis algorithms, it is difficult to evaluate the existing algorithms for wearable single-lead ECG analysis, due to the lack of such an ECG database with heartbeat annotations. Therefore, building a wearable single-lead ECG database is of great significance to the feasibility study of the computer-aided automated arrhythmia diagnosis for daily wearable ECG signals.

**Figure 2:**
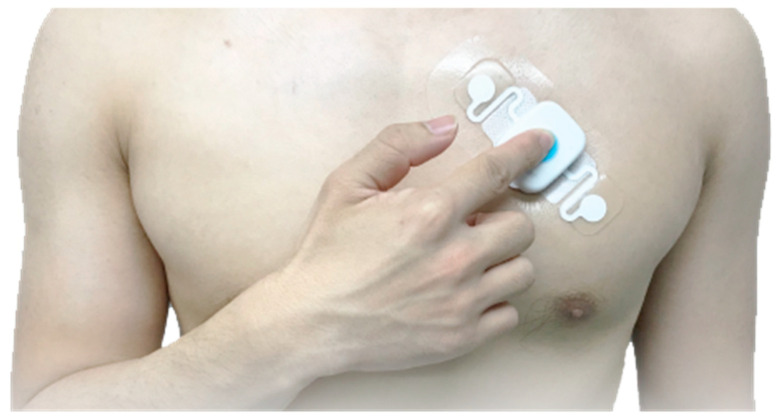
The electrode location for ECG monitoring using the CarePatch™ ECG patch.

The CarePatch™ ECG patch database (CPPDB) contains 123 30-min wearable single-lead recordings of 123 patients’ daily ECG data collected from July 2017 to February 2020. Since certain arrhythmias attack occasionally and transiently, these patients were requested to wear the CarePatch™ ECG patch continuously for as long as possible within 7 days (the patch is cleared for 7-day continuous ECG monitoring). The device collected the wearable non-standard lead ECG signals at the recommended electrode location [32] as shown in Figure 2. The sample rate of the ECG signals is 256 Hz, and the resolution is 12-bit. As a result, the average duration of the raw ECG data was 104.10 h, and we further selected 30-min continuous ECG fragments from each of these raw data to compose a dataset with as many arrhythmia heartbeats as possible. The basic information of the CPPDB is listed in Table 3. Due to the privacy issue, one patient did not register the gender and 29 patients did not register their age.

**Figure 3:**
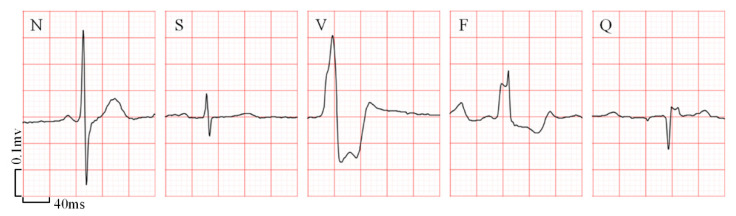
The samples of five heartbeat categories in the CPPDB. The background stands for a standard ECG paper and a small box represents 40 ms on the interval and 0.1 mV on the amplitude.

Consistent with the MITDB dataset, the heartbeats in these recordings were annotated as N, S, V, F, and Q, five classes, by a consensus committee of three expert cardiologists. The examples of the samples in each heartbeat class are shown in Figure 3, where the background stands for standard ECG paper and the small box represents 40 ms on the interval and 0.1 mV on the amplitude. Table 4 shows the number of each heartbeat category in the database and the training set as well as the test set, respectively. In the future, the CPPDB would be made partially available to other researchers with the same ethical standards.

## 4. Results

### 4.1. Experimental Settings

The system configuration of the test platform was Matlab R2018a and Python 3.6.8 running on Ubuntu 16.4.7. The server featured Intel^®^ Xeon^®^ CPU E5-2678 v3 (12/24 cores/threads, 2.5/3.1 GHz base/turbo) and 112 GB 2400 MHz DDR4 RAM. In addition, the experiment involving the CNN for comparison used two Nvidia^®^ GeForce^®^ RTX 2080Ti GPUs (1350/1454 MHz base/boost with 11GB GDDR 6 VRAM).

During the ECG preprocessing stage, we optimized the filter parameters by the grid search method. The $a_{median}$, $f_{l}$, $f_{u}$, $f_{order}$, and $beta_{Kaiser}$ were set to 0.9, 0.05 Hz, 40 Hz, 341, and 4.538, respectively. Then we trained our model using the toolkit in the XGBoost python module. The “objective” refers to the learning task and corresponding target, which was set to “multi:softmax”, and the “eval_metric” defines the evaluation index used for the verification, which was set to “merror” to use the multi-classification error rate. The “min_child_weight” and “scale_pos_weight” refer to the sum of the weight of the smallest sample in the leaf node and the sample imbalance correction coefficient, respectively, which were both set to “1”.

For other parameters, we also ran the grid search to find the optimal values, as shown in Table 5. Estimators are the basic decision tree as the “n_estimators” refers to the number of trees. Generally, the more basic decision trees, the less likely the model is overfitted. However, too many decision trees will also result in a more complicated model. The “max_depth”, “subsample”, and “colsample_bytree” are three important tree-based parameters. A greater max depth of the tree usually results in a more specific machine learning model. Yet an overly large “max_depth” may also cause overfitting. Reducing the proportion of random sampling of subtrees could enhance the generalization ability of the model. Meanwhile, the parameter “colsample_bytree” is the number of the feature randomly selected for the training of each tree expressed as a fraction of features. Two parameters regarding the learning process should be optimized as well. The parameter “reg_alpha” refers to the L1 regularization term on weights and could help to reduce overfitting. Finally, ‘eta’ is analogous to the learning rate, which makes the model more robust by shrinking the weights on each step. The detailed tuning process of these parameters is presented in Appendix A.

To sum up, the final selected parameters used for model training are illustrated in Table 6. All parameters were optimized using the MITDB. Furthermore, once these parameters were set, they were directly applied to the training using the CPPDB without searching again for a fair comparison with other methods.

### 4.2. Experimental Results

**Figure 4:**
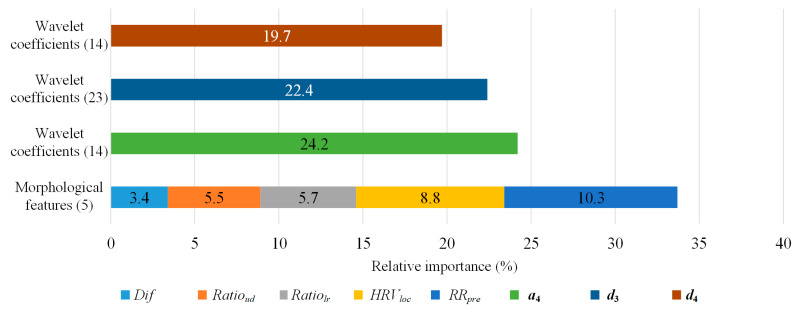
The relative importance of the features on the MITDB. The number in the brackets is the dimension of features.

We first analyzed the impact of the proposed morphological features on the XGBoost method by deriving the relative importance based on the weight of features, which is the number of times a feature is used to split the data across all trees. As shown in Figure 4, although the morphological features only had five dimensions, they played an important role in heartbeat classification as each feature contributed 6.74% of the feature weight on average. Meanwhile, the average contribution of the feature weight for wavelet features was 1.3%. Therefore, the morphological features had a significant effect on the model.

**Figure 5:**
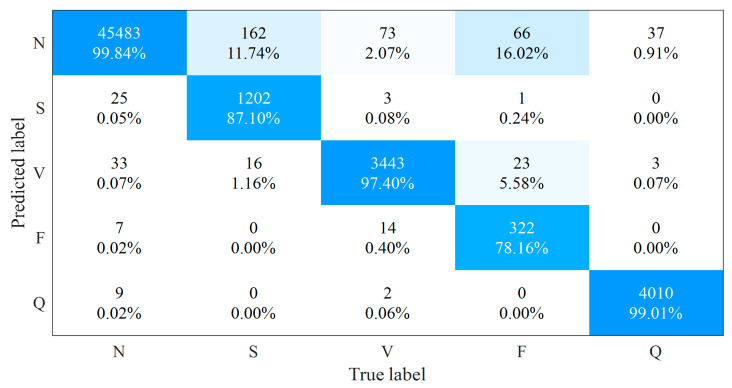
The confusion matrix of the proposed method on the MITDB.

Figure 5 presents the confusion matrix of our method on the MITDB and Table 7 gives the averaged Acc and detailed results of Se, +P, as well as F1 for each type of heartbeats among different methods. Our method outperforms other state-of-the-art methods regarding the averaged classification accuracy and achieves the highest performance for the remaining three indicators in N-type and Q-type heartbeats among these methods.

For the S-type and V-type heartbeats, our method is better than [16,17,18,19] for sensitivity, precision, and *F*1 score. The sensitivity of the 2-lead ResNet method [20] is higher than that of ours by 2.2% and 0.4% for S-type and V-type heartbeats, respectively. However, the corresponding precision of our method is higher than that of [20] by 2.6% and 0.2%, where the *F*1 scores are very close to each other. For the F-type heartbeats, although CNN methods [18,19] have a higher sensitivity than our methods, their precision is significantly lower; the 2-lead ResNet method [20] has a higher sensitivity and *F*1 score than our method, while our method has higher precision. Nevertheless, different from other methods, the ResNet method in [20] used the 2-lead ECG signals to analyze the heartbeat, where combining the heartbeat classification probabilities provided by the corresponding heartbeats in the two leads of the MITDB should improve the heartbeat analysis performance. However, when facing single-lead ECG signals, the lack of additional lead information would affect the performance of the multi-lead ECG-based method and limit its application in wearable ECG monitoring scenarios.

**Figure 6:**
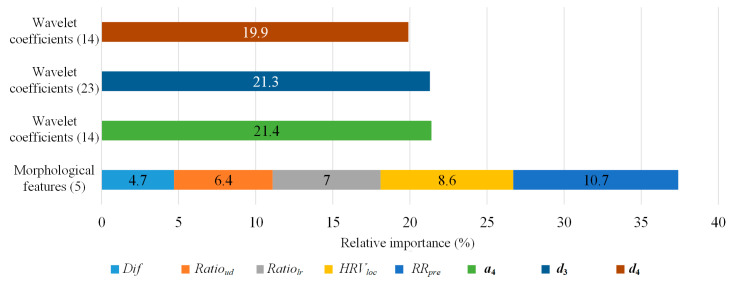
The relative importance of the features on the CPPDB. The number in the brackets is the dimension of features.

**Figure 7:**
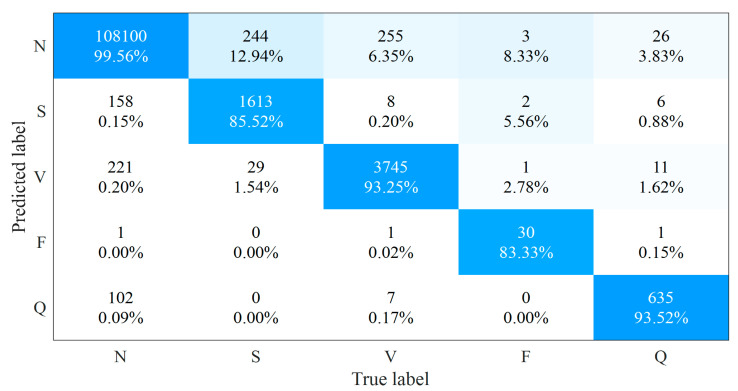
The confusion matrix of the proposed method on the CPPDB.

To further evaluate methods for the wearable single-lead ECG signals, we trained our method on the self-collected wearable single-lead ECG databases, i.e., the CPPDB. Since we collected these ECG recordings in a daily, unlimited-use environment, the signals could well reflect the real-world wearing situation, and they were introduced to complicated noise situation as well. As can be seen in Figure 6, the relative importance of the morphological features saw a further rise by a total of 3.7% when facing the wearable single-lead ECG signals, suggesting these features could better distinguish different types of heartbeats from ECG signals in daily monitoring condition and help maintain the robustness of the algorithm for wearable single-lead ECG. The confusion matrix of the proposed method on the CPPDB is shown in Figure 7. Since the amount of the F-type heartbeats in CPPDB is significantly less than that of the MITDB, the F-type classification in CPPDB could benefit from smaller intra-class differences and result in improved performance. Regarding the heartbeat classification of the remaining four classes, there existed varying degrees of performance loss due to more complicated daily environmental noises and more heartbeat patterns that wearable devices can obtain in long-term monitoring.

We also reproduced the open-source methods above [16,17] on the CPPDB. Notice that the algorithm parameters of our method were determined using the MITDB to avoid data leakage in parameter tuning for a fair comparison with the provided open-source models. The results in Table 8 indicate that all three methods suffered from the realistic fair signal quality of the wearable single-lead ECG with the accuracy loss. Still, our method outperforms the hand-crafted feature-based SVM method [16] and the deep-learning-based CNN method [17] for all four performance indicators in the average of the five heartbeat classes.

Table 9 listed the running time of the three methods tested on the baseline MITDB. The running time was calculated by first obtaining the start time and end time of the corresponding code of different methods, and then subtracting the two time points. The MATLAB code was realized through the combination of function “tic” and function “toc”, and the Python code was realized through the function “time”. Notice that the CNN method [17] required GPU for training, while the SVM method [16] and our method only relied on CPU. We could observe that our method cost less training time than the other two methods without the GPU acceleration, where the feature extraction stage occupied about 90% of the duration. For the test phase, the ECG analysis time of our method was also the least among these three methods with less than a second for 30-min single-lead ECGs with CPU only, and the proportion of the feature extraction time consumed decreased to about 60%. On the other hand, the CNN method spent an average time of 8.55 s for a 30-min single-lead ECG analysis, even though it benefited from the parallel computing of GPU.

We also discussed the performance of our method under long-term monitoring and whether our method can analyze input signals in real time. Specifically, we measured the inference time of our method when the duration of the input signal is different. The five ECG recordings with durations from 30 min to 360 min were selected from the CarePatch™ ECG patch database. For each recording, we conducted five repeated experiments on the same hardware environment. It can be seen from Table 10 that the inference time basically increases linearly with the duration of the input signal, which is consistent with the time complexity O(n) of our method, indicating that our method could process ECG signals under long-term monitoring conditions.

For the “real-time”, there remain no unified provisions on whether the heartbeat classification method is real-time. It is generally believed that when the collected ECG segment (the duration is usually 500 ms or 1 s) was input to the heartbeat classification method, if the method can obtain the results before the input of the next ECG segment, the method is considered to be real-time. We used the test set DS2 of MITDB to conduct the real-time experiment. We divided 22 ECG recordings into 500 ms or 1 s segments, respectively, and input them into our trained method to obtain the average inference time. The inference time in Table 11 indicates that our method could classify the heart beats in real time in the test environment.

## 5. Discussion

### 5.1. Pros and Cons of the XGBoost Method

Compared with traditional machine learning methods, the proposed XGBoost-based beat-level ECG analysis further improved the accuracy of the heartbeat classification by mining the morphological characteristics and wavelet coefficient characteristics in the heartbeat fragments. The XGBoost algorithm avoids model redundancy as well as the over-fitting problem, and reduces the feature dimension required for the model training by adding a regular term about the model to the objective function; in addition, since XGBoost aims at parallelization, the processing is optimized with the feature blocks preferentially generated at the feature level, so that the feature blocks in the iterative process could be called repeatedly, and the gain calculation of each feature could be derived in parallel through multi-threading. Therefore, compared with the beat-level ECG analysis method based on deep learning, the XGBoost method has a more concise model description and lower training cost, which would enable future high-accuracy personalized ECG models and algorithm deployment in mobile computing scenarios.

Since the feature vector generation of the algorithm proposed in this paper is based on each heartbeat fragment, this method is more feasible for the short-term ECG analysis, while the long-term one based on the heartbeat feature extraction would introduce high computational costs for heartbeat segmentation and feature deriving. Furthermore, S and V heartbeats as well as F and V heartbeats have similar characteristics, hence the handcrafted features might limit the performance of the classifier for these two groups of different heartbeats. New heartbeat features could be introduced for the above categories, such as the indicator of the P wave existence, etc., to improve the accuracy of the beat-level ECG analysis.

### 5.2. The Wearable Single-Lead ECG Database

The results showed that all the methods suffered from performance loss on the self-collected wearable single-lead ECG database. Although various algorithms have been proposed and evaluated on widely used open-source ECG databases like MITDB and achieved high accuracies, the studies on automated ECG analysis are still limited by the lack of ECG data, resulting in decreasing performance in realistic clinical applications and a massive proofreading workload of ECG interpretations for medical staff. Most of the publicly available ECG databases contained few subjects, i.e., less than 200 persons, and were collected in the resting condition [33] with relatively good signal quality. Not to mention, the channels of the ECG recordings were mostly based on certain leads of the standard 12-lead ECG system. Therefore, these ECG databases could be the baseline for algorithm evaluation yet are not feasible enough for smart model training to process single-lead ECGs, especially for the ambulatory ECG signals collected by wearable devices with a variety of forms of non-standard ECG leads.

We built the wearable single-lead ECG database using the clinical patch-type wearable ECG device. The recordings in the CPPDB referred to the non-standard lead and signals were collected in people’s daily life. Hence, the CPPDB could be used to better evaluate the performance of the algorithm on wearable ECG signals. However, our database currently has an imbalance problem for the certain heartbeat and segment classes, which would affect the classification performance of algorithms for these categories. To sum up, the construction of a public wearable single-lead ECG database is significant to the development of the automated wearable single-lead ECG analysis, yet still has a long way to go regarding the status quo.

## 6. Conclusions

In this paper, we propose novel morphological features for the XGBoost beat-level ECG analysis to achieve a robust heartbeat classification for everyday wearable single-lead ECGs. The widely used public ECG database and a self-collected wearable single-lead ECG database were applied for methods evaluation. The results showed that our method outperformed other state-of-the-art methods regarding the accuracy of both databases. As clinical wearable ECG monitoring devices are getting mature, both highly robust beat-level wearable ECG analysis methods and new ECG signal analysis modes like segment-based ECG classification should be further explored to adapt to wearable ECG signal acquisition modes, e.g., the immediate real-time and ultra-long-term ECG monitoring.

## Figures and Tables

**Table 1:** Evaluation scheme of the MITDB.

Dataset	Index of MITDB Recordings											Amount
DS1	101	106	108	109	112	114	115	116	118	119	122	22
	124	201	203	205	207	208	209	215	220	223	230	
DS2	100	103	105	111	113	117	121	123	200	202	210	22
	212	213	214	219	221	222	228	231	232	233	234	

The recordings 102, 104, 107, and 217 were excluded since they are paced ECGs.

**Table 2:** Evaluation scheme of the MITDB heartbeat samples.

AAMI Class	MITDB Class	Sum	DS1	DS2
N	NOR, LBBB, RBBB, AE, NE	91,043	45,486	45,557
S	AP, aAP, NP, SP	2791	1411	1380
V	PVC, VE	7235	3700	3535
F	fVN	797	385	412
Q	P, fPN, U	8002	3952	4050
Total amount		109,868	54,934	54,934

**Table 3:** The basic statistics of the samples from the CPPDB.

Variables	Statistics		Remarks
Participant summary	123 patients	55 males, 67 females	1 not registered
Age, years (mean ± SD ^1^)	49 ± 23	min: 1, max: 93	29 not registered
Raw data duration, hours (mean ± SD ^1^)	104.10 ± 56.74	min: 1.64, max: 176.95	-

^1^ “SD” refers to the standard deviation.

**Table 4:** Data distribution of the CPPDB

Class	Total Amount	Training Set	Test Set
N	217,165	108,583	108,582
S	3772	1886	1886
V	8031	4015	4016
F	73	37	36
Q	1358	679	679
Sum	230,599	115,300	115,299

**Table 5:** Adjustment process of important parameters of XGBoost.

Parameter	Initial Value	Tuning Range	Step
n_estimators (coarse-grained) n_estimators (fine-grained)	-	10–300	10
	-	160–180	1
max_depth	5	2–20	1
subsample	0.8	0.05–1	0.05
colsample_bytree	44/56	1/56–1	1/56
reg_alpha	0	0–0.05	0.005
eta	0.17	0.01–0.3	0.01

**Table 6:** The optimized parameters of XGBoost.

Parameter	Value
n_estimators	164
max_depth	11
subsample	0.6
colsample_bytree	48/56
reg_alpha	0.01
eta	0.2

**Table 7:** The performance comparison with the state-of-the-art methods on the MITDB

**Ref.**	Acc (%)	N			S			V			F			Q		
		Se (%)	+P (%)	F1 (%)	Se (%)	+P (%)	F1 (%)	Se (%)	+P (%)	F1 (%)	Se (%)	+P (%)	F1 (%)	Se (%)	+P (%)	F1 (%)
[16]	94.47	95.9	98.2	-	78.1	49.7	-	94.7	93.9	-	12.4	23.6	-	-	-	-
[17]	96.58	97.6	98.5	-	76.8	74.0	-	93.8	92.4	-	79.6	62.4	-	-	-	-
[18]	98.00	99.4	98.6	99.0	75.6	96.8	84.9	93.8	95.1	94.4	85.8	65.7	74.4	-	-	-
[19]	98.41	99.5	99.0	99.2	77.9	87.7	82.3	94.5	95.7	95.1	82.1	83.7	82.9	98.5	99.3	98.9
[20]	99.06	99.7	99.3	99.5	89.3	95.0	92.0	97.8	97.7	97.7	80.4	92.0	85.8	98.9	99.7	99.3
Ours	99.14	99.8	99.3	99.6	87.1	97.6	92.1	97.4	97.9	97.6	78.2	93.9	85.3	99.0	99.7	99.4

“-” means the corresponding value was not provided originally or could not be calculated.

**Table 8:** The performance comparison on the CPPDB.

Ref.	Acc (%)	Se (%)	+P (%)	F1 (%)
[16]	94.39	90.75	88.94	89.20
[17]	95.31	87.32	84.17	85.83
Ours	98.68	91.03	91.89	91.39

**Table 9:** The running time comparison for different methods on the MITDB.

Ref.	Time of the Training Phase ^1^ (min)			Time of the Test Phase ^1,2^ (s)		
	Feature Extraction	Classifier Training	Total Time	Feature Extraction	Classifier Inference	Total Time
[16]	23.36 ± 0.56	4.69 ± 0.01	28.05 ± 0.58	0.51 ± 0.00	0.63 ± 0.00	1.15 ± 0.00
[17]	-	-	28.74 ± 0.35	-	-	8.55 ± 0.02
Ours	22.11 ± 0.11	2.42 ± 0.02	24.53 ± 0.13	0.49 ± 0.00	0.33 ± 0.00	0.82 ± 0.00

^1^ The data are presented in the form of “average value ± standard deviation”. ^2^ The test time was the average analysis duration for each lead of MITDB recordings that contains 30-min ECG.

**Table 10:** The inference time for different duration of input signals.

ECG Recording Duration	Time of the Method Inference ^1^ (s)		
	Feature Extraction	Classifier Inference	Total Time
30min	0.52 ± 0.08	0.32 ± 0.04	0.84 ± 0.14
60min	0.99 ± 0.11	0.59 ± 0.07	1.59 ± 0.19
90min	1.59 ± 0.13	0.91 ± 0.06	2.50 ± 0.20
120min	2.12 ± 0.13	1.22 ± 0.05	3.34 ± 0.19
180min	3.28 ± 0.16	1.79. ± 0.07	5.08 ± 0.26
360min	6.72 ± 0.21	3.56 ± 0.09	10.30 ± 0.34

^1^ The data are presented in the form of “average value ± standard deviation”.

**Table 11:** The inference time for different duration of ECG segments.

Duration	Time of the Method Inference ^1^ (ms)		
	Feature Extraction	Classifier Inference	Total Time
500 ms	26.34 ± 0.11	16.25 ± 0.05	42.62 ± 0.17
1 s	33.21 ± 0.12	17.28 ± 0.04	51.07 ± 0.19

^1^ The data are presented in the form of “average value ± standard deviation”.

**Figure A1:**
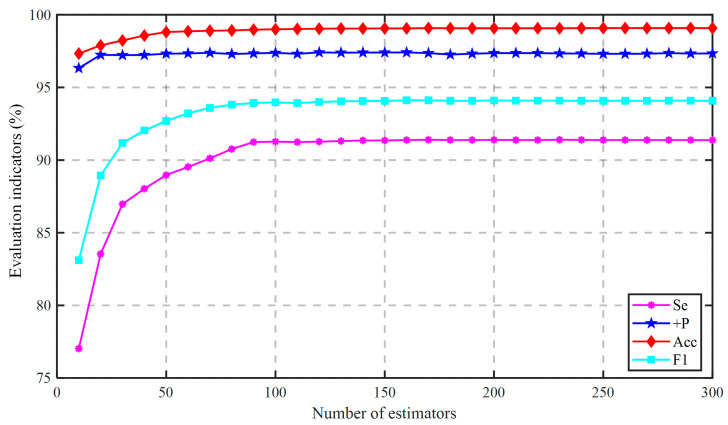
The coarse-grained tuning of the “n_estimators”.

**Figure A2:**
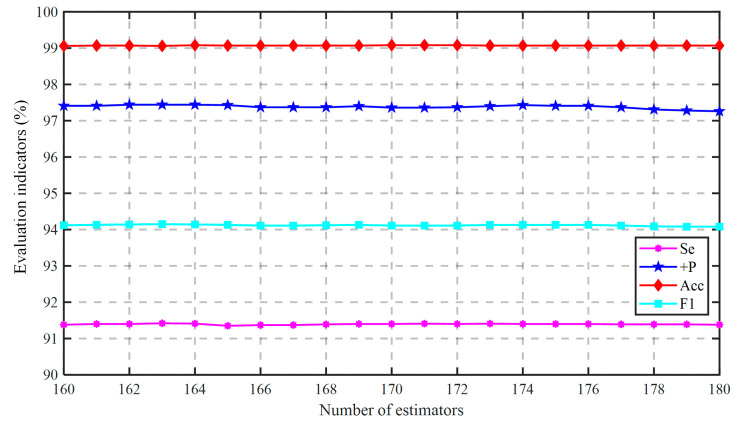
The fine-grained tuning of the “n_estimators”.

**Figure A3:**
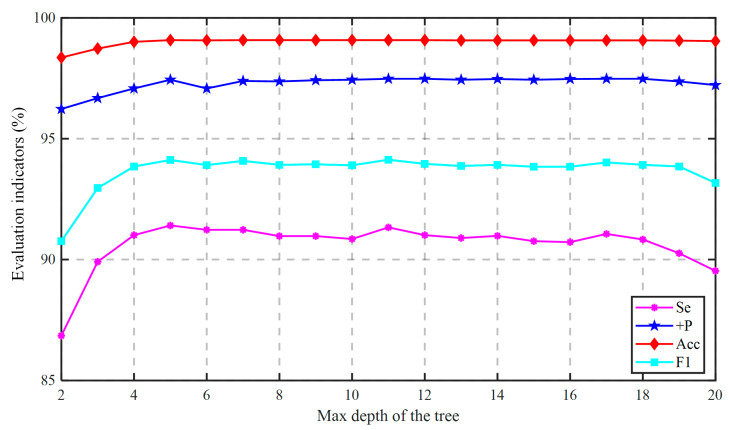
The performance change with the “max_depth”.

**Figure A4:**
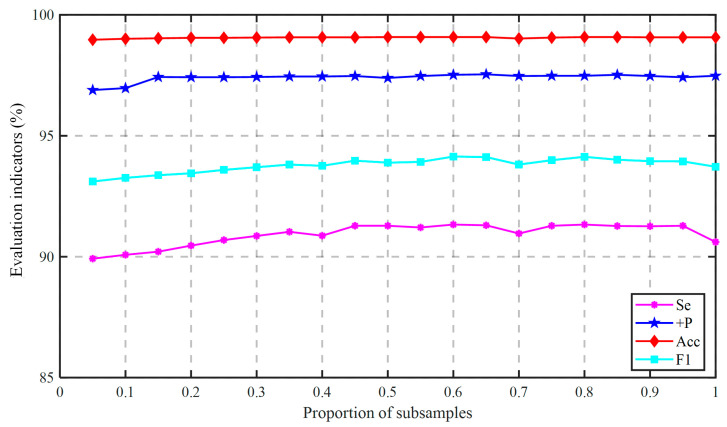
The performance change with the “subsample”.

**Figure A5:**
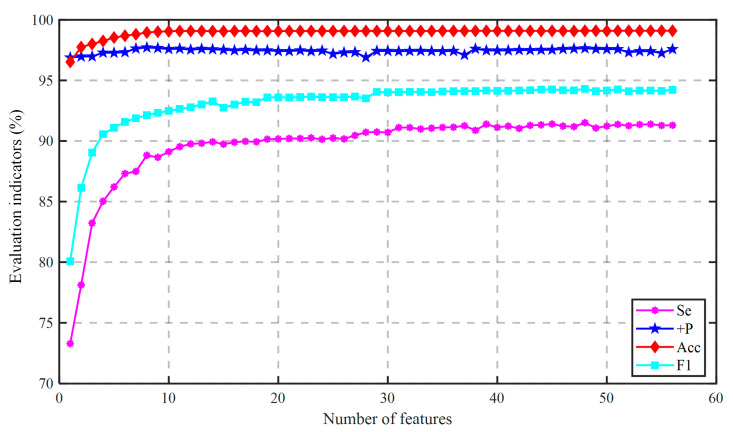
The performance change with the “colsample_bytree”.

**Figure A6:**
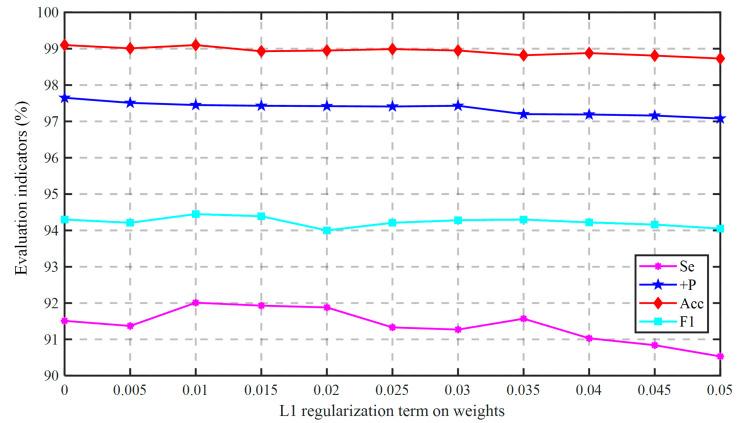
The performance change with the “reg_alpha”.

**Figure A7:**
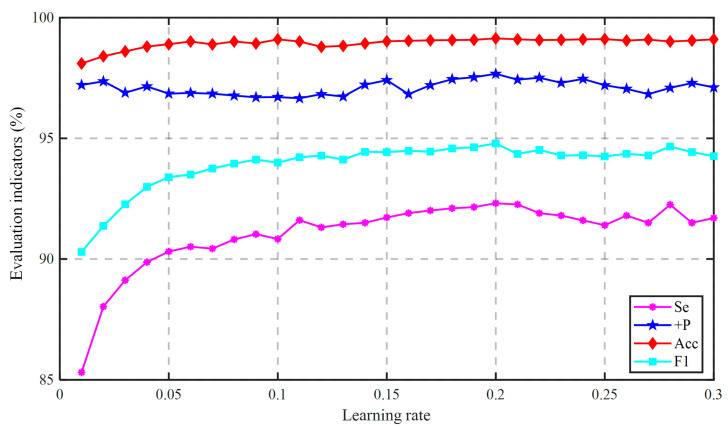
The performance change with the “eta”.

## Data Availability

The MIT-BIH Arrhythmia Database employed in our study are openly available in [https://www.physionet.org/physiobank/database/mitdb/, accessed on 4 August 2021] at [10.13026/C2F305].

## Appendix A

In this section, we show the optimization process of parameters in detail. The number of estimators is usually the first parameter to be tuned in XGBoost, and it is better to be below 300. We applied the hierarchical search method to screen the “n_estimators” efficiently. We first set the tuning range to 10–300 with the granularity of 10 in the coarse-grained stage. As shown in Figure A1, we got the highest performance when the number of estimators was 170. Then we started the fine-grained search with a limited tuning range of 160–180 and a reduced granularity of 1. It can be observed in Figure A2 that the comprehensive performance (the highest Acc with a larger Se, +P, and F1) was optimal when the number of estimators was 164.

After fixing the number of estimators, we could adjust tree-based parameters, i.e., the “max_depth”, “subsample”, and “colsample_bytree”. Figure A3 showed that all the four indicators increased at the beginning as the max depth increased, and Acc obtained its max results when the max depth was 5. Hereafter, the indicators started to fluctuate with the further increase of max depth due to the effect of overfitting. As a result, Acc, +P, and F1 reached their optimal value as the max depth was 11.

We then used a sampling scale ranging from 0.05 to 1, and a step size of 0.05 in the training model. As shown in Figure A4, when the sampling ratio was 0.1, all four indicators exceeded 90%, and Acc reached the highest value of 99.08% when the sampling ratio was 0.6, and the other three indicators were also competitive. Meanwhile, it could be seen in Figure A5 that as the number of features increased, the performance indicators showed an increasing trend. Acc, Se, and F1 reached their optimized values and +P was very close to the max, when the “colsample_bytree” got 48/56.

The next step was to fine-tune the regularization parameters. Figure A6 presents the experimental results corresponding to different “reg_alpha” values, from which we could see that when the value is 0.01, Acc, Se, and F1 achieved their highest value, and +P was competitive as well. Lastly, we should tune “eta”. Figure A7 shows the effect of eta on the algorithm. All four indicators obtained the highest value when “eta” was 0.2.
